# Analysis of Erosive Wear in Pipe Elbows and Biomimetic Protection Strategies

**DOI:** 10.3390/biomimetics11050336

**Published:** 2026-05-11

**Authors:** Zhenjiang Wei, Chengchun Zhang, Hongzhi Sun, Chun Shen, Meihong Gao, Meihui Zhu

**Affiliations:** 1 Department of Mechanical and Electronic Engineering, Heze University, Heze 274015, China; 2 Heze Shuanglong Metallurgical Machinery Co., Ltd., Heze 274015, China; 3 Key Laboratory of Bionic Engineering, Ministry of Education, Jilin University, Changchun 130022, China

**Keywords:** erosion resistance, liquid–solid two-phase flow, biomimetic design, shell ribbed structure

## Abstract

Erosive wear in pipe elbows subjected to liquid–solid two-phase flow is a major cause of material degradation and service failure in industrial piping systems. In this study, erosion characteristics of pipe elbows were investigated through erosion mapping experiments and numerical simulations. The effects of flow velocity and particle diameter on erosion location and intensity were analyzed. Erosion was found to be mainly concentrated on the outer wall of the elbow within the angular range of 10° to 90°, and both erosion intensity and affected area increased with increasing particle diameter and flow velocity. Dean vortices were shown to play an important role in particle transport and erosion distribution, especially for small particles. Inspired by the ribbed morphology of shells, a biomimetic elbow was further designed and evaluated through an orthogonal numerical study considering flow velocity, particle diameter, rib number, and rib diameter. The results indicate that the ribbed structure can effectively improve erosion resistance by altering particle trajectories, reducing particle impact probability, and dissipating kinetic energy through low-velocity rotating flow between adjacent ribs. This finding provides useful inspiration for addressing erosive wear problems in engineering applications.

## 1. Introduction

Erosive wear refers to the phenomenon wherein fluid or solid particles impact the surface of an object at a specific velocity and angle [1,2], resulting in material loss. This process is typically accompanied by complex physical and mechanical interactions, making it one of the prevalent forms of material failure in industrial applications. Erosive wear is widely observed across various sectors, including fluid-driven agricultural machinery [3], energy exploration [4,5], aerospace [6,7], and transportation systems [8,9]. For instance, in agricultural equipment, soil particles carried by fluids strike machinery surfaces; in the energy sector, high-velocity sand-laden fluids in pipelines abrade pipe walls; and in aerospace, aircraft engine blades may suffer erosion due to ingested dust particles. In these scenarios, erosive wear not only reduces equipment longevity but also poses potential safety risks [10,11], rendering it a critical consideration in engineering design and material selection.

Erosive wear is a common failure mode in engineering systems involving particle-laden flows and is governed by complex interactions among particle velocity, impact angle, material properties, and flow conditions. Classical studies, including those of Finnie [12,13], Bitter [14], and Hutchings [15,16], established the basic theoretical framework for understanding cutting wear, deformation wear, and impact-induced material removal. These studies indicate that erosion is a multiscale process controlled by both particle dynamics and wall response, which provides an important theoretical basis for erosion prediction and protection design.

Through billions of years of evolution, biological systems have developed unique structural features that provide abundant inspiration and guidance for solving engineering problems [17,18]. Ren et al. [19,20] found that the surface morphology and multilayered structure of desert lizards can significantly enhance the erosion resistance of biological materials, and elucidated the underlying anti-erosion mechanisms based on stress wave theory. Han et al. [21] reported that Tamarix exhibits excellent resistance to erosion in severe wind-blown sand environments, which is primarily attributed to its surface crack structures that can disperse impact stresses and inhibit crack propagation. Wang et al. [22] demonstrated that conch shells can inspire the optimization of hydraulic valve cores, where the incorporation of biomimetic anti-erosion structures markedly improves their erosion resistance. Our research group [23] conducted CFD–DEM numerical simulations on shell structures and found that their ribbed morphology can effectively protect the surface by reducing particle impact velocity and collision frequency. Although rib-based anti-erosion strategies have been reported in previous studies, most of them focused on relatively simple exposed surfaces or local components. In contrast, the present study addresses erosive wear in pipe elbows under liquid–solid two-phase flow, where erosion is jointly affected by curvature-induced secondary flow, particle inertia, and particle–wall interaction in a confined flow passage. Therefore, the anti-erosion role of ribbed structures in elbows cannot be directly inferred from previous rib-based studies and requires dedicated analysis under elbow flow conditions.

In piping systems, elbows are among the most vulnerable components to erosive wear because abrupt changes in flow direction intensify particle impingement and secondary-flow effects [24,25,26,27]. Studies have demonstrated that the erosion rate at elbow sections can exceed that of straight pipe segments by more than 50 times [28]. In practical engineering contexts, such as oil and gas field development, the degradation of pipeline elbows due to erosive wear often manifests over several years, resulting in prolonged experimental cycles that are challenging to replicate in laboratory settings [29,30].

To address this issue, researchers use numerical simulations to model the flow field distribution of the continuous phase and the particle trajectories of the discrete phase at the pipeline elbow to calculate the extent of erosion wear. Common numerical simulation methods include Computational Fluid Dynamics–Discrete Phase Model (CFD-DPM) and Computational Fluid Dynamics–Discrete Element Method (CFD-DEM). In CFD-DPM, the continuous phase is solved using CFD, while the discrete phase is typically handled using the Lagrangian particle tracking method. This method calculates the particle trajectories and usually solves the particle-to-particle contact and collision processes. Ransmark et al. [31] used CFD-DPM to predict particle erosion in high-pressure homogenizer valves and systematically studied the influence of valve geometry, operating conditions, and particle characteristics on erosion behavior. Khan et al. [32] used this method to simulate particle-laden flow behavior and identify the critical erosion zones of centrifugal pump blades. Their results indicated that the severity of erosion is related to turbulence intensity and particle impacts. CFD-DEM also uses CFD to solve the continuous phase, but the discrete phase is handled using the Discrete Element Method (DEM). Compared to DPM, DEM further explicitly calculates the interactions, collisions, friction, rebound, and packing behavior between particles and between particles and the wall. Hong et al. [33] adopted the CFD-DEM coupled calculation method to study the internal flow field characteristics and erosion rate of a needle valve under gas–solid two-phase flow conditions. The results showed that as the particle diameter increases, the maximum erosion rate of the valve increases. When the valve opening is 0.5, the effect of suppressing the erosion rate is optimal at high flow velocities. In engineering applications, CFD-DPM is used to simulate dilute flows with a particle volume fraction less than 10%, while CFD-DEM is used for dense flows with a particle volume fraction greater than this value [34].

In the present study, CFD–DPM was employed to simulate the flow behavior of particles inside the pipe in order to investigate the erosion problem in the pipe elbow. By analyzing the erosion patterns on the inner surface of elbows caused by liquid–solid two-phase flows under varying flow velocities and solid particle characteristics, this research elucidates the underlying mechanisms of erosion. Validation is achieved through erosion mapping experiments, ensuring the reliability of the findings. Inspired by the anti-erosion function of shell ribbed structures, a biomimetic pipe elbow was designed. Erosion behavior of the biomimetic elbow was systematically investigated under varying particle diameters, flow velocities, and rib diameters. An orthogonal experimental design was employed to determine the optimal combination of parameters for erosion resistance. Finally, the underlying anti-erosion mechanisms of the biomimetic elbow were elucidated through detailed flow field analysis. These results provide a reference for erosion protection strategies and the optimization of elbow designs in the context of oil and gas field development, contributing to enhanced durability and performance of piping systems in such applications.

## 2. Erosion Experimental Apparatus and Methodology

### 2.1. Erosion Test Pipe Elbow Model

**Figure 1:**
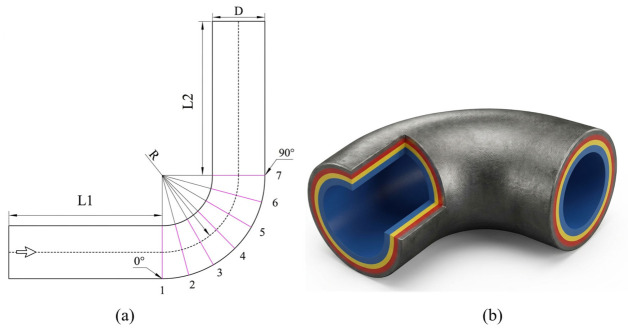
(**a**) Geometry of the elbow; (**b**) elbow test specimen.

The geometric model of the elbow pipeline is illustrated in Figure 1a. The pipe diameter (D) is 26 mm, and the curvature radius (R) is 38 mm. The upstream and downstream lengths are set to three times the pipe diameter, i.e., L1 = L2 = 78 mm. The fluid enters through the lower pipe and exits through the upper pipe.

In engineering practice, pipeline erosion may take several years or even decades to manifest, making it challenging to replicate in a laboratory setting. This study applies latex paints of different colors along the thickness direction of the pipeline’s inner wall to qualitatively observe the location and intensity of erosion at the elbow. A standard seamless welded elbow is selected as the test specimen, made of carbon steel, with a pipe diameter of 26 mm and a curvature-to-diameter ratio (R/D) of 1.5. Three latex paints—red, yellow, and blue—are chosen and mixed with gypsum powder at a mass ratio of 2:1. An appropriate amount of diluent is added and stirred evenly to produce three distinct colored coatings. A brush is used to apply the red coating evenly onto the inner surface of the elbow, with a thickness just sufficient to cover the surface completely. The elbow is then placed in a hot air drying oven set at 150 °C and heated with ventilation for 1 h to rapidly dry the coating. This process is repeated to sequentially apply the yellow and blue coatings, resulting in an elbow with three distinct colored layers, as shown in Figure 1b. It should be noted that the multilayer latex paint + gypsum method used in this study is essentially a qualitative erosion-mapping technique based on coating removal. Its main purpose is to identify erosion-prone regions and compare the relative erosion distribution with numerical simulations, rather than to directly quantify the actual erosion loss of the carbon-steel substrate. In the present work, the coating thickness was not directly measured, because the coating layers were applied primarily to ensure complete and distinguishable surface coverage for qualitative visualization.

### 2.2. Equipment and Experimental Conditions

**Figure 2:**
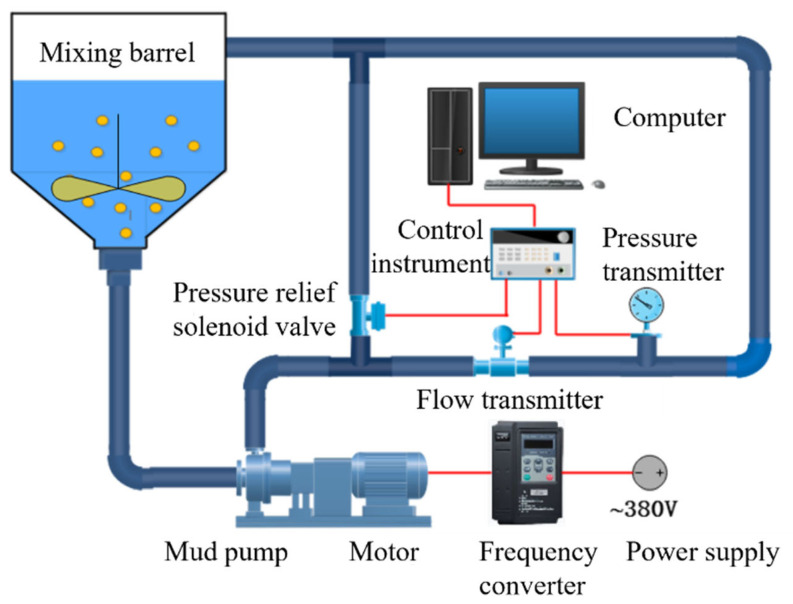
Erosion experimental equipment.

The schematic diagram of the equipment system for the erosion wear test is shown in Figure 2. An electric motor drives a slurry pump to circulate the particle-laden fluid from a mixing tank through the test elbow, inducing erosive wear on the elbow. A frequency converter adjusts the motor’s speed, thereby altering the flow velocity of the liquid within the pipeline. Pressure and flow transmitters measure the pressure and flow rate inside the pipeline, respectively. When the pressure in the pipeline system exceeds a predetermined threshold, a pressure relief solenoid valve automatically opens to ensure the safety of the experiment.

**Figure 3:**
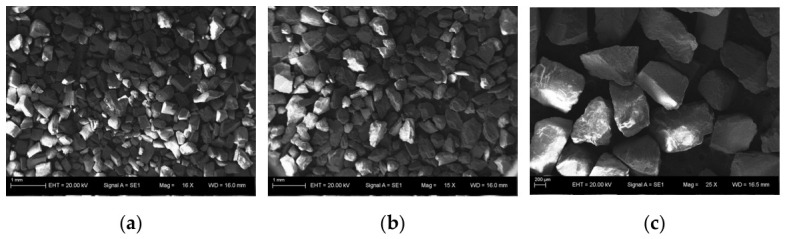
Surface morphology of the quartz sand used in this study: (**a**) 80–120 mesh, (**b**) 40–60 mesh, (**c**) 20–40 mesh.

Four flow velocities—2.5, 3.0, 3.5, and 4.0 m/s—are investigated in combination with three quartz sand particle size ranges, 20–40 mesh (1.0 mm), 40–60 mesh (0.5 mm), and 80–120 mesh (0.2 mm), as shown in Figure 3. In total, 12 experimental conditions are evaluated, with each test conducted for 1 h.

## 3. Numerical Models and Methods

### 3.1. Discrete Phase Model

In the numerical simulation of liquid–solid two-phase flow, the liquid phase is modeled by solving the Navier–Stokes equations based on the SST k-ω turbulence model [35,36,37,38]. This approach accounts for the transport of turbulent shear stress and incorporates limitations in the turbulence formulation to prevent the overprediction of turbulent viscosity. The continuous phase influences the discrete phase particles through drag forces and turbulence effects, while the particles, in turn, affect the fluid by reducing the mean momentum and turbulence intensity, establishing a two-way coupling process. The particle trajectories are obtained by integrating the particle motion equations in a Lagrangian coordinate system. It is assumed that the particles are independent of one another, neglecting inter-particle collisions and particle fragmentation. The governing equations for the particles are derived from Newton’s second law:

(1) $$\left\{\begin{array}{l} \frac{du_{p}}{dt}=F_{D}(u-u_{p})+\frac{g_{x}(\rho_{p}-\rho )}{\rho_{p}}\\ F_{D}=\frac{18\mu }{\rho_{p}d_{p}^{2}}+\frac{C_{D}Re}{24}\\ Re=\frac{\rho d_{p}\left|u_{p}-u\right|}{\mu }\\ C_{D}=\frac{24}{Re_{sph}}(1+b_{1}R{e_{sph}}^{b_{2}})+\frac{b_{3}Re_{sph}}{b_{4}+Re_{sph}} \end{array}\right.$$

(2) $$\left\{\begin{array}{l} b_{1}=\exp(2.3288-6.4581f+2.4486f^{2})\\ b_{2}=0.0964+0.5565f\\ b_{3}=\exp(4.905-13.8944f+18.4222f^{2}-10.2599f^{3})\\ b_{4}=\exp(1.4681+12.2584f-20.7322f^{2}+15.8855f^{3}) \end{array}\right.$$

where *ρ* represents the fluid density, *u* denotes the fluid phase velocity, *u_p_* is the particle velocity, and *F_D_*(*u* − *u_p_*) represents the drag force per unit mass of the particle. *μ* denotes the dynamic viscosity of the fluid, *ρ_p_* is the particle density, and *d_p_* represents the particle diameter. *Re* denotes the relative Reynolds number, while *b*_1_, *b*_2_, *b*_3_, and *b*_4_ are coefficients associated with *f*. The parameter *f* is defined as the shape factor, which is the ratio of the surface area of a sphere with the same volume as the particle to the actual surface area of the particle. The parameter *Re_sph_* represents the Reynolds number of a sphere with the same volume as the particle.

### 3.2. Erosion Model

The erosion model is defined by Equation (3), with its parameters determined through multiple erosion tests on Inconel 718 alloy [39]. The reliability of this model has been validated in the literature [40]. This model can accurately describe the wall thickness loss distribution on the surface of elbows and effectively characterize the particle flow field distribution in solid–liquid two-phase flow erosion models.

(3) $$\left\{\begin{array}{l} ER=C{\left(BH\right)}^{-0.59}F_{s}\nu_{p}^{n}F\left(\theta \right)\\ F\left(\theta \right)=5.40\theta -10.11\theta^{2}+10.93\theta^{3}-6.33\theta^{4}+1.42\theta^{5} \end{array}\right.$$

where *ER* represents the erosion rate with units of kg/(m^2^·s), while *C* is a constant related to the properties of the target material, with a value of 2.17 × 10^−7^. The exponent of impact velocity, *n*, is taken as 2.41. The particle shape factor, *F_s_*, is 1 for sharp-edged particles, 0.2 for spherical particles, and 0.53 for particles with intermediate shapes. *BH* denotes the Brinell hardness of the target material, which is taken as 160, measured in MPa. The parameter *θ* represents the impact angle of the particles, and *v_p_* is the impact velocity with units of m/s. The function *F*(*θ*) is an empirical function of the impact angle derived from experimental data fitting.

### 3.3. Geometric Model and Boundary Conditions

The continuous-phase medium is water at ambient temperature, with other parameters listed in Table 1. During the extraction and transportation of petroleum, crude oil often contains suspended solids such as particles and clay, with the particle content potentially reaching up to 5% by volume [41]. Khan et al. [42] used numerical simulations and found that a mass fraction of 10% (4% by volume) was more likely to observe erosion phenomena. To balance both engineering practicalities and the erosion phenomena, the particle mass fraction in this study is set to 10%. The mass concentration of particles is uniformly set to 10%. The inlet and outlet are assigned escape conditions (Escape), while the wall surfaces are set to reflection conditions (Reflect). In the present model, drag and inertial effects were treated as the dominant factors governing particle transport in the elbow, while the pressure gradient force and lift force were neglected for model simplification. For discretization, second-order upwind schemes are applied to momentum and turbulent kinetic energy equations. The Discrete Phase Model (DPM) is used for solid particles, and pressure–velocity coupling is handled using the SIMPLE algorithm [43]. A two-way coupling approach is adopted for the discrete phase, where the particle trajectories are computed every 10 continuous-phase iterations. The momentum information obtained from the discrete phase is then incorporated into the continuous phase calculations until a steady-state solution is achieved. The simulation experiments are divided into four groups based on different continuous-phase flow velocities. Each group includes three trials, varying the diameter of the discrete-phase particles.

### 3.4. Mesh Independence

**Figure 4:**
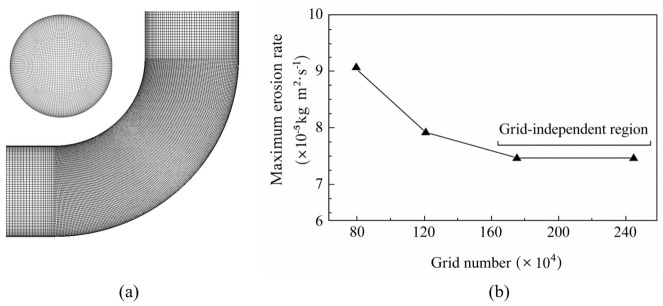
(**a**) Elbow mesh; (**b**) curve of maximum erosion rate and number of meshes.

The type and size of the grid significantly affect both the computational accuracy and the overall computational effort. An O-Block topology method was used to divide the pipeline grid, with a finer mesh applied to the bend section, as shown in Figure 4a. Performing a grid independence study allows for determining the appropriate mesh density for the problem, ensuring the accuracy of the numerical simulation. Wilson [44,45] recommended that the number of nodes on each edge of the grid should increase by a factor of 2. Following this method, simulations are conducted for a continuous phase flow velocity of 3 m/s and a particle diameter of 0.5 mm. The relationship between the maximum erosion rate and the grid density is shown in Figure 4b. With a lower number of grids, the maximum erosion rate fluctuated irregularly as the grid count increased. However, after reaching 1.74 million grids, the maximum erosion rate stabilized. To minimize computational errors to the greatest extent within the available computational capacity, a grid count of 1.74 million was chosen.

## 4. Erosion Analysis of the Pipe Elbow

### 4.1. Comparison Between Experimental and Numerical Simulation Results

**Figure 5:**
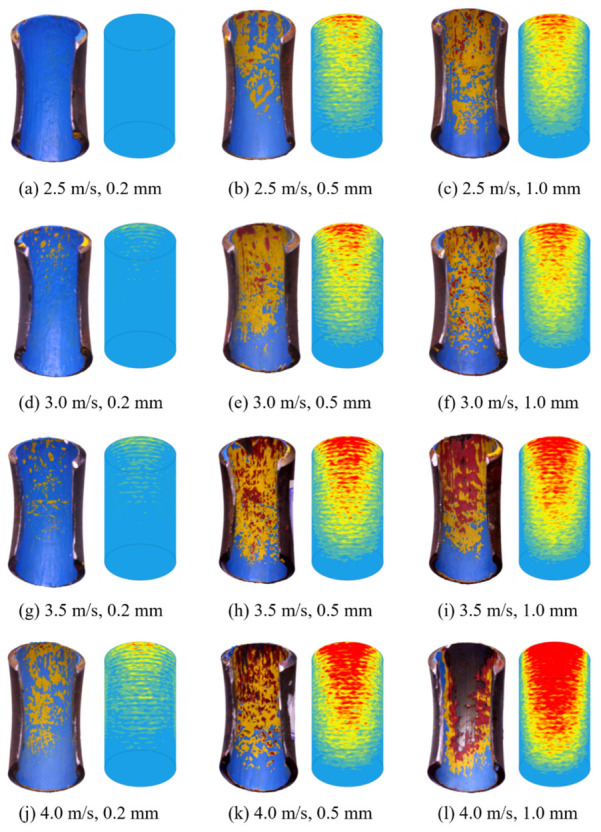
Test elbow (left) and numerical simulation (right) erosion contours.

Experiments and numerical simulations are performed using flow velocity and particle diameter as the controlling parameters. The experimentally observed erosion patterns and the total erosion contour maps obtained from the numerical simulations are presented in Figure 5. As shown, the predicted erosion locations and intensities are in good agreement with the experimental results across all test conditions. With increasing particle diameter and flow velocity, the erosion region expands from the central area of the outer bend toward the surrounding regions, while the maximum erosion consistently occurs at the center of the outer curvature.

### 4.2. Influence on the Distribution of Erosion Locations

The Stokes number [46,47] is a dimensionless parameter that describes the motion characteristics of particles in a fluid. It is defined as the ratio of the particle momentum response time to the fluid characteristic time, representing the relative magnitude of particle inertia to drag force. The equation is given as

(4) $$S_{t}=\frac{u(\rho_{p}-\rho )d_{p}^{2}}{18\mu D}$$

where *u* represents the water flow velocity, *ρ_p_*, and *ρ* denote the densities of the particle and the fluid, respectively, *μ* is the fluid viscosity, *D* is the pipe diameter, and *d_p_* is the particle diameter. As indicated by Equation (4), the Stokes number is positively correlated with particle density *ρ_p_*, particle diameter *d_p_*, and flow velocity *u*, but negatively correlated with fluid viscosity *μ* and pipe diameter *D*. Therefore, a larger Stokes number indicates that particle motion is more strongly dominated by inertia and less able to follow the local fluid streamlines. In contrast, when the Stokes number is small, particles are more readily entrained by the carrier flow and are more susceptible to secondary-flow structures inside the elbow.

**Figure 6:**
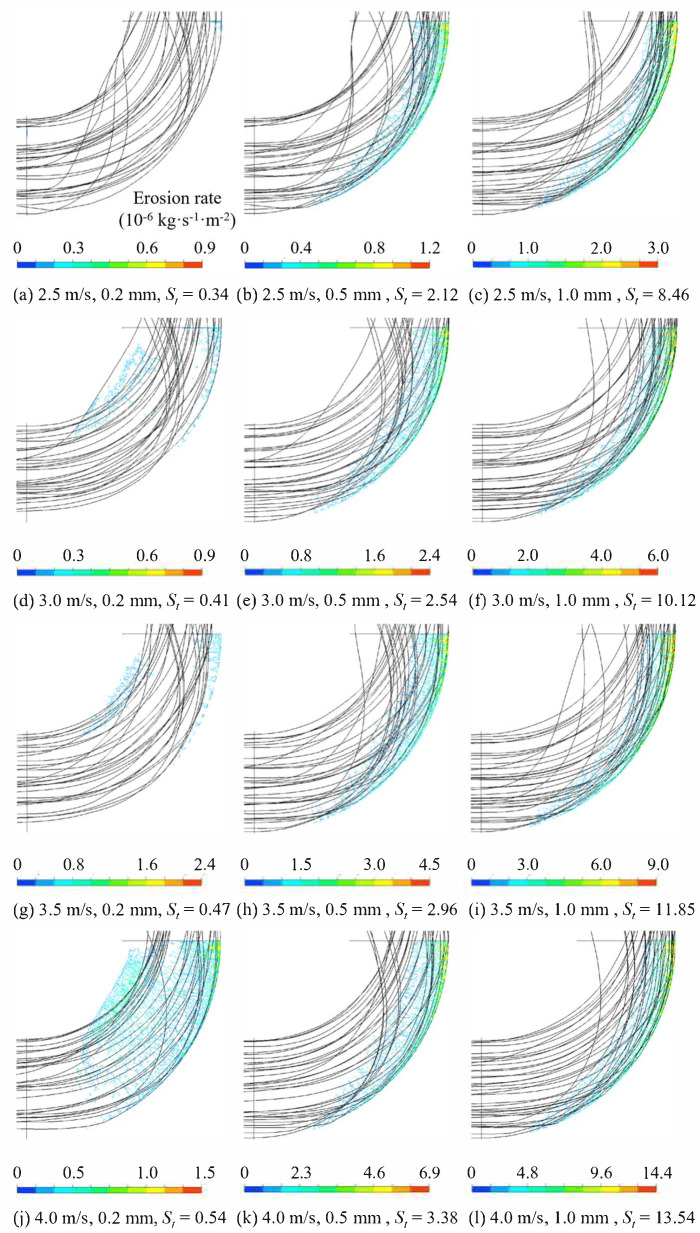
Particle trajectories and erosion-rate contours of the smooth elbow under different particle diameters and flow velocities. The color bar indicates erosion rate.

The Stokes number influences the particle trajectory, and the Stokes numbers and motion trajectories under various conditions are shown in Figure 6. As the particle diameter increases, the erosion intensity on the inner side gradually decreases, while that on the outer side progressively increases. This phenomenon occurs because larger particle diameters correspond to higher Stokes numbers, enhancing the particles’ ability to traverse the flow field. Consequently, due to inertial effects, particles have an increased likelihood of colliding with the outer side of the bend, leading to a progressive concentration of the erosion region at the outermost part of the pipe elbow as the particle diameter increases.

**Figure 7:**
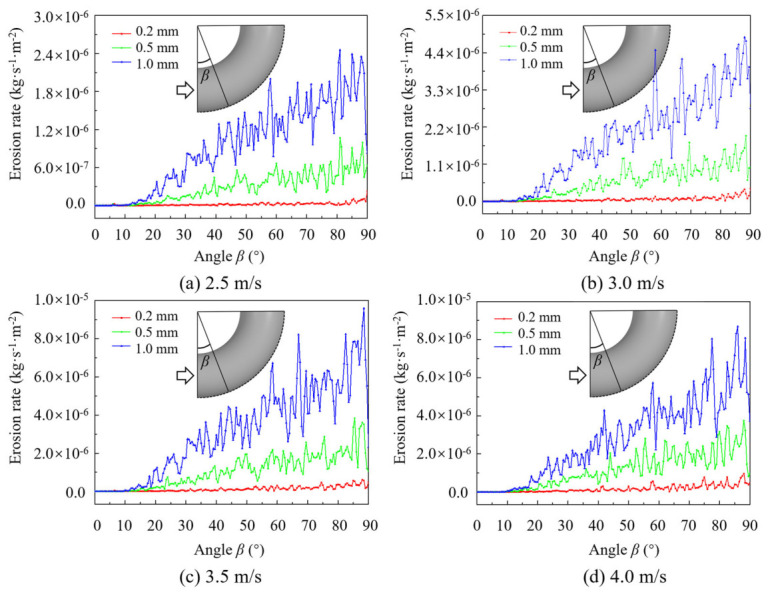
Curve of erosion rate of elbows with angle.

To more accurately investigate the relationship between particle velocity, diameter, and erosion rate, a centerline is drawn along the outer side of the bend, and 180 data points are uniformly selected. The erosion rate at each point is extracted, and the relationship between the elbow angle and the erosion rate is plotted, as shown in Figure 7. The variation pattern of erosion rates with particle diameter at different velocities is generally consistent. Within the range of 0° to 10°, as the liquid–solid two-phase flow first enters the elbow, the turbulence intensity and velocity gradient of the flow field are relatively lower compared to the high-angle region. At this stage, only a small number of particles impact the outer wall, and no significant erosion is observed. Between 10° and 90°, the erosion rate increases approximately linearly with the angle, reaching its maximum at 90°. Moreover, as the particle diameter increases, the linear growth coefficient also increases. This is because a larger elbow angle results in a higher number of particle–wall collisions. At the same angle, larger particles possess greater energy, leading to stronger impact forces upon collision with the wall surface.

### 4.3. Effect of Dean Vortices on Erosion

**Figure 8:**
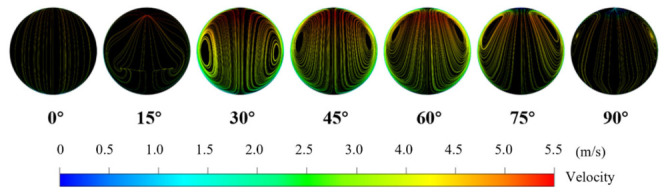
Cross-sectional streamline patterns at different elbow angles (0°, 15°, 30°, 45°, 60°, 75°, 90°) for a continuous-phase velocity of 4 m/s. The color bar indicates velocity magnitude (m/s).

At the same particle size, the erosion rate increases with velocity. However, when the particle size is small (*d* = 0.2 mm), the erosion area in the elbow tends to be evenly distributed across the entire high-angle region, as shown in Figure 6g. To investigate the effect of the internal continuous phase flow velocity distribution on erosion intensity, seven sections were selected at 15° intervals from 0° to 90° along the elbow with a continuous phase velocity of 4 m/s for streamline analysis, as shown in Figure 8. When the fluid first enters the pipe elbow, the streamlines in the cross-section of the elbow are uniform. At the 15° position of the elbow, the streamlines curve, and a rudimentary form of Dean vortices [48,49] appear near the outer side of the elbow. At 30°, the Dean vortices are fully formed, and as the elbow angle increases, the center of the Dean vortices moves from the outer side toward the inner wall. At 60°, the vortex center moves outward, and at the elbow exit, the streamlines at the section center become flatter, and the range of the Dean vortices shrinks, leading to the dissipation of the Dean vortex phenomenon. During this process, smaller particles, owing to their lower Stokes number and stronger flow-following capability, are more readily influenced by the Dean vortices, resulting in erosion on both sides of the elbow.

**Figure 9:**
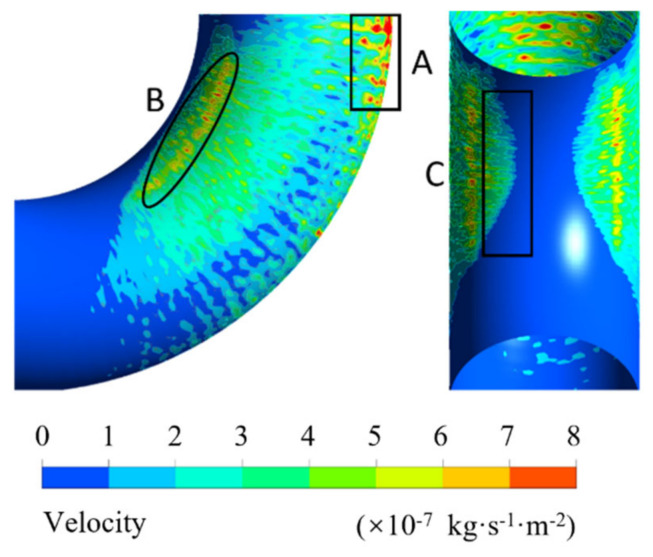
Erosion contour of the elbow at a flow velocity of 4 m/s and a particle diameter of 0.2 mm, showing the main erosion zone (A), secondary erosion zone (B), and erosion boundary (C). The color bar indicates erosion rate.

Erosion analysis is shown in Figure 9. Erosion phenomena begin at 15° in the elbow and intensify as the elbow angle increases, forming the main erosion zone at point A and a secondary erosion zone at point B. The erosion at point A is caused by particles with a certain velocity impacting the outer side of the elbow due to their inertial forces. Under the influence of the Dean vortices, some particles collide with friction against the outer side of the elbow, leading to erosion at point B. Point C represents the erosion boundary on the inner side of the elbow, with symmetrical distribution on both sides. The boundary line is shaped like a parabola with the opening oriented in the left–right direction. Measurements show that the closest point of the parabola to the inner side of the elbow occurs at 64.17°. This phenomenon aligns with the motion trajectory of the Dean vortex center.

## 5. Erosion Resistance Performance of Biomimetic Elbows

### 5.1. Biomimetic Elbow Model

**Figure 10:**
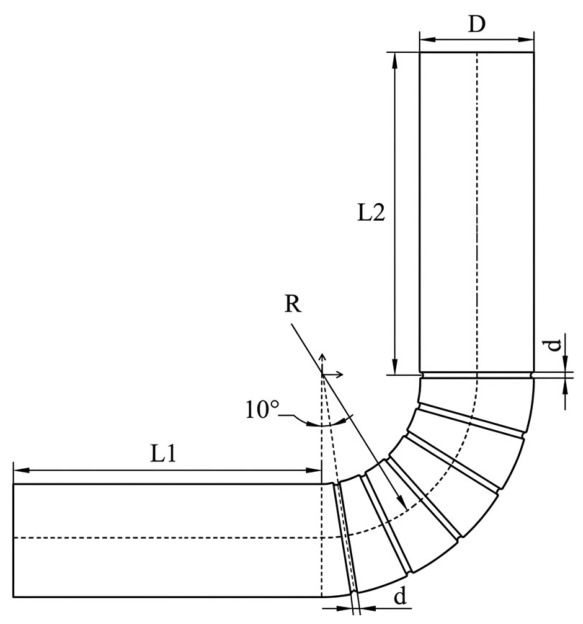
Geometric model of the biomimetic elbow pipe.

Scallops living in sediment-laden environments are continuously subjected to the impact of solid particles. The cylindrical ribbed structures on their surfaces play a crucial role in enhancing erosion resistance [23], providing a novel design strategy for mitigating erosive wear in pipe elbows. Based on the experimental and numerical simulation results presented above, erosive wear is predominantly concentrated within the angular range of 10° to 90°. Therefore, semi-cylindrical ribs oriented perpendicular to the incoming flow direction are arranged within this region. The rib cross-section has a diameter of *d*, and a total of *n* ribs are distributed, as illustrated in Figure 10.

### 5.2. Numerical Analysis of Erosive Wear in the Biomimetic Elbow

The numerical simulations primarily investigate the effects of biomimetic rib characteristic dimensions, rib arrangement number, particle velocity, and particle diameter on the erosive wear of the biomimetic elbow. Four factors are selected, each with three levels: A—flow velocity (3 m/s, 5 m/s, 7 m/s), B—particle diameter (0.2 mm, 0.5 mm, 0.8 mm), C—number of ribs (6, 8, 10), and D—rib diameter (0.5 mm, 1 mm, 1.5 mm). The levels of each factor are listed in Table 2. Factor A is determined based on the safe flow velocity for oil transportation in petrochemical industries, with moderately increased values to extend the prediction range. The particle diameter in factor B is selected within the same range as that used in the numerical simulations and experiments for the smooth elbow. The biomimetic rib arrangements for factors C and D are illustrated in Figure 10.

In this study, an L9(3^4^) orthogonal array was used as a screening design to reduce the number of numerical simulations and to evaluate the main effects of flow velocity, particle diameter, rib number, and rib diameter on the relative erosion rate. Interaction effects between factors were not considered in the present design in order to control the computational cost. Therefore, the orthogonal analysis in this study should be interpreted as a main-effect-based comparative analysis rather than a full statistical optimization including interaction effects.

The erosive wear characteristics of the elbow are evaluated using the area-weighted average method and the relative erosion rate. The area-weighted average erosion rate is obtained by summing the products of the erosion rate and the corresponding unit surface area over all surface elements, and then dividing by the total surface area. The relative erosion rate is calculated using the following expression:

(5) $$ER=\frac{ER_{biomimetic}}{ER_{smooth}}$$

In the equation, *ER* denotes the relative erosion rate, which is a dimensionless quantity; *ER_biomimetis_* represents the area-weighted average erosion rate of the biomimetic elbow, and *ER_smooth_* represents the area-weighted average erosion rate of the smooth elbow.

### 5.3. Range Analysis

The relative erosion rate and the corresponding range analysis results obtained from the numerical simulations are summarized in Table 2. As shown, variation in the number of ribs results in the largest change in relative erosion rate (*R_C_* = 0.1029), followed by rib diameter (*R_D_* = 0.0463), flow velocity (*R_A_* = 0.0378), and particle diameter (*R_B_* = 0.0355). Therefore, the order of influence of structural parameters and operating conditions on the relative erosion rate (ER) of the biomimetic elbow is rib number (C) > rib diameter (D) > flow velocity (A) > particle diameter (B).

It should be noted that only range analysis was performed in the present orthogonal design. Since the L9(3^4^) array assigns the available degrees of freedom mainly to the four investigated factors, no independent residual error term is available for a rigorous ANOVA without additional repeated or supplementary simulations. Therefore, *p*-values and statistical significance levels were not reported. The present orthogonal analysis is mainly used to identify the relative importance of the main factors and to guide the selection of a favorable ribbed-structure configuration.

By comparing the values of each factor at different levels, the optimal parameter combination is determined to be A_3_B_2_C_3_D_3_, corresponding to a flow velocity of 7 m/s, a particle diameter of 0.5 mm, a rib number of 10, and a rib diameter of 1.5 mm. This optimal combination is not included in the original experimental design, which is a reasonable outcome in orthogonal experimental analysis. In terms of erosion resistance, the rib-related parameters have a more pronounced effect on the relative erosion rate than flow velocity and particle diameter. Within the selected parameter range of this numerical study, the biomimetic elbow can therefore be expected to maintain favorable erosion resistance under varying operating conditions.

### 5.4. Erosion Resistance Mechanism of the Biomimetic Elbow

**Figure 11:**
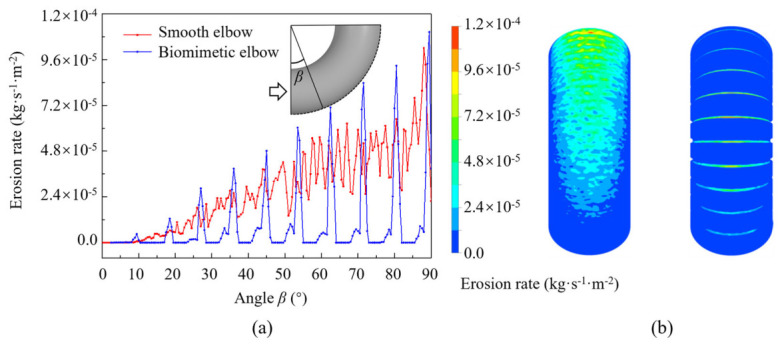
(**a**) Curve of erosion rate of elbows with angle; (**b**) erosion distribution in the elbow pipe.

A comparison of the erosion rates between the biomimetic elbow (A_3_B_2_C_3_D_3_) and a smooth elbow is presented in Figure 11. Using Equation (5), the relative erosion rate of the biomimetic elbow is 0.8971, indicating that the erosion resistance of the biomimetic elbow is improved by 10.29% compared with that of the smooth elbow. For the smooth elbow, the erosion intensity and distribution are consistent with the previously described results, primarily occurring within the 10° to 90° range, with the erosion rate increasing as the bend angle increases. In contrast, for the biomimetic elbow, erosion is predominantly concentrated on the rib structures, and the erosion region decreases as the bend angle decreases. The erosion intensity on the elbow wall is significantly lower than that of the smooth surface, whereas the erosion intensity on the ribs is higher than that at the corresponding locations in the smooth elbow.

**Figure 12:**
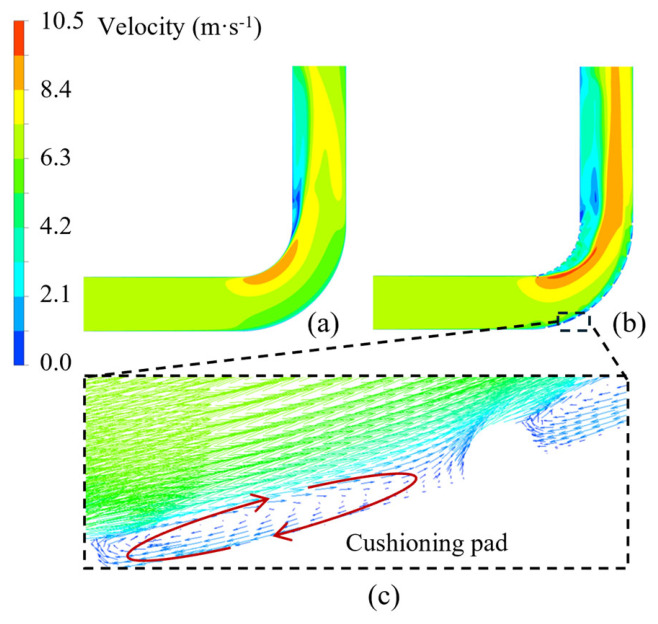
Velocity contour distributions: (**a**) smooth elbow; (**b**) biomimetic elbow; (**c**) local enlargement of (**b**).

Figure 12 presents the velocity vector distribution in the vicinity of the ribs. It can be observed that, due to the obstruction of the ribs to the flow field, a distinct and stable vortical region forms between adjacent ribs. The flow velocity in this region is significantly lower than that in the central region of the elbow, generally below approximately 2 m/s. This flow pattern effectively acts as a “cushioning pad” formed by rotating fluid between the ribs. First, the velocity in the central region of the elbow increases, and owing to the drag interaction between the fluid and particles, particles are more likely to pass through the bend, reducing their probability of entering the inter-rib regions. In addition, the presence of ribs disrupts the stable flow field near the pipe wall, generating low-velocity rotating flow. This results in higher turbulence intensity compared to that over a smooth wall, causing many particles that would otherwise impact the wall to deviate in trajectory and preferentially migrate toward the core flow region. Meanwhile, the low-velocity rotating flow acts to attenuate the impact energy of the particles by dissipating a substantial portion of their kinetic energy, thereby weakening their erosive effect on the ribs.

Overall, the present results reveal a distinct structure–property–performance relationship for the biomimetic elbow. The ribbed structural design, especially the rib number and rib diameter (structure), changes the near-wall flow characteristics and particle transport behavior, including the formation of low-velocity vortical regions, particle trajectory deflection, reduced particle–wall impact probability, and kinetic energy dissipation (property). These effects collectively lead to a lower relative erosion rate and enhanced erosion resistance of the elbow (performance).

## 6. Conclusions

In this study, the erosive wear behavior of pipe elbows under liquid–solid two-phase flow was investigated through erosion experiments and numerical simulations, and a biomimetic anti-erosion strategy inspired by shell ribbed structures was proposed. The main conclusions are as follows:

(1) The experimental erosion patterns were in good agreement with the numerical simulation results, confirming the reliability of the adopted CFD-based erosion model for predicting erosion location and intensity in pipe elbows.
(2) Erosion in the smooth elbow was mainly concentrated on the outer wall within the angular range of 10° to 90°, and the maximum erosion rate consistently occurred near the center of the outer curvature. Both erosion intensity and erosion area increased with increasing particle diameter and flow velocity.
(3) Particle diameter significantly affected erosion distribution through the Stokes number. Larger particles exhibited stronger inertial effects and were more likely to impact the outer wall of the elbow, leading to a more concentrated erosion zone. For smaller particles, Dean vortices played an important role in particle transport, causing erosion to extend over a wider region.
(4) Inspired by the anti-erosion function of shell ribbed morphology, a biomimetic elbow with semi-cylindrical ribs was designed. Within the scope of the present orthogonal simulation design, the influence of different factors on the relative erosion rate followed the order: rib number > rib diameter > flow velocity > particle diameter. The favorable parameter combination was identified as A_3_B_2_C_3_D_3_, under which the area-weighted average erosion rate was reduced by 10.29% compared with that of the smooth elbow under the same numerical conditions.
(5) The erosion-reduction mechanism of the biomimetic elbow is mainly associated with rib-induced modification of the near-wall flow field. The ribs generated low-velocity vortical structures between adjacent ribs, which altered particle trajectories, reduced particle–wall impact probability, and dissipated part of the particle kinetic energy. These effects contributed to improved erosion resistance of the elbow wall under the conditions considered in this study.

From an industrial application perspective, the proposed biomimetic ribbed elbow provides a potential passive protection strategy for mitigating erosion in particle-laden pipeline systems, such as those used in petroleum extraction, slurry transportation, and other liquid–solid two-phase flow conditions. By arranging ribbed structures in the erosion-prone region of the elbow, particle trajectories can be regulated and near-wall impact intensity can be reduced without introducing additional active control devices. Nevertheless, the present study was conducted under selected laboratory and numerical conditions. Further long-term erosion tests, quantitative substrate material-loss measurements, and validation under realistic industrial flow conditions are required before large-scale engineering application.

## Figures and Tables

**Table 1:** Parameter setting for simulation calculation.

	Fluid	Particle	Pipe
*ρ* [kg/m^3^]	998.2	2650	7800
*μ* [kg/m∙s]	1.003 × 10^−3^	/	/
*d_p_* [mm]	/	0.2, 0.5, 1.0	/
*F_s_*	/	0.75	/
*u* [m/s]	2.5, 3.0, 3.5, 4.0	2.5, 3.0, 3.5, 4.0	/
Flow rate [kg/s]	/	0.133, 0.159, 0.186, 0.212	/
Turbulence intensity	5%	/	/
Hydraulic diameter [m]	0.026	/	/

**Table 2:** Orthogonal experimental results of erosion simulation in the biomimetic elbow.

Num.	Experimental Scheme				Relative Erosion Rate (ER) *y_i_*
	Flow Velocity A (m/s)	Particle Diameter B (mm)	Number of Ribs C	Rib Diameter D (mm)	
1	1 (3)	1 (0.2)	1 (6)	1 (0.5)	1.1089
2	1 (3)	2 (0.5)	2 (8)	2 (1.0)	0.9695
3	1 (3)	3 (0.8)	3 (10)	3 (1.5)	0.9416
4	2 (5)	1 (0.2)	2 (8)	3 (1.5)	0.9623
5	2 (5)	2 (0.5)	3 (10)	1 (0.5)	0.9532
6	2 (5)	3 (0.8)	1 (6)	2 (1.0)	1.0527
7	3 (7)	1 (0.2)	3 (10)	2 (1.0)	0.9473
8	3 (7)	2 (0.5)	1 (6)	3 (1.5)	0.9893
9	3 (7)	3 (0.8)	2 (8)	1 (0.5)	0.9701
$\bar{y_{j1}}$	1.0067	1.0062	1.0503	1.0107	Factors Order: C, D, A, B Optimal combination: A_3_B_2_C_3_D_3_
$\bar{y_{j2}}$	0.9894	0.9707	0.9673	0.9898	
$\bar{y_{j3}}$	0.9689	0.9881	0.9474	0.9644	
$R_{j}$	0.0378	0.0355	0.1029	0.0463	

## Data Availability

The original contributions presented in this study are included in the article. Further inquiries can be directed to the corresponding authors.
